# Transgenic Zebrafish Reveal Tissue-Specific Differences in Estrogen Signaling in Response to Environmental Water Samples

**DOI:** 10.1289/ehp.1307329

**Published:** 2014-01-14

**Authors:** Daniel A. Gorelick, Luke R. Iwanowicz, Alice L. Hung, Vicki S. Blazer, Marnie E. Halpern

**Affiliations:** 1 Department of Embryology, Carnegie Institution for Science, Baltimore, Maryland, USA; 2 Fish Health Branch, U.S. Geological Survey, Kearneysville, West Virginia, USA

## Abstract

Background: Environmental endocrine disruptors (EEDs) are exogenous chemicals that mimic endogenous hormones such as estrogens. Previous studies using a zebrafish transgenic reporter demonstrated that the EEDs bisphenol A and genistein preferentially activate estrogen receptors (ERs) in the larval heart compared with the liver. However, it was not known whether the transgenic zebrafish reporter was sensitive enough to detect estrogens from environmental samples, whether environmental estrogens would exhibit tissue-specific effects similar to those of BPA and genistein, or why some compounds preferentially target receptors in the heart.

Methods: We tested surface water samples using a transgenic zebrafish reporter with tandem estrogen response elements driving green fluorescent protein expression (5xERE:GFP). Reporter activation was colocalized with tissue-specific expression of ER genes by RNA *in situ* hybridization.

Results: We observed selective patterns of ER activation in transgenic fish exposed to river water samples from the Mid-Atlantic United States, with several samples preferentially activating receptors in embryonic and larval heart valves. We discovered that tissue specificity in ER activation was due to differences in the expression of ER subtypes. ERα was expressed in developing heart valves but not in the liver, whereas ERβ2 had the opposite profile. Accordingly, subtype-specific ER agonists activated the reporter in either the heart valves or the liver.

Conclusion: The use of 5xERE:GFP transgenic zebrafish revealed an unexpected tissue-specific difference in the response to environmentally relevant estrogenic compounds. Exposure to estrogenic EEDs *in utero* was associated with adverse health effects, with the potentially unanticipated consequence of targeting developing heart valves.

Citation: Gorelick DA, Iwanowicz LR, Hung AL, Blazer VS, Halpern ME. 2014. Transgenic zebrafish reveal tissue-specific differences in estrogen signaling in response to environmental water samples. Environ Health Perspect 122:356–362; http://dx.doi.org/10.1289/ehp.1307329

## Introduction

Estrogens are small molecules that influence organ formation and function (Deroo and Korach 2006). Estrogens bind to and activate receptors in the cytosol, which then travel to the nucleus and directly regulate gene expression. Multiple estrogen receptor (ER) genes are present in vertebrates, such as the *Esr1* and *Esr2* genes in mice (coding for ERα and ERβ proteins, respectively) and the *esr1*, *esr2a*, and *esr2b* genes in zebrafish (coding for ERα, ERβ1, and ERβ2 proteins, respectively). Exposure to environmental endocrine disruptors (EEDs) that bind to ERs are associated with increased risk of cancers and abnormal reproductive tract formation in mammals and fish (Ma 2009). Because ERs are expressed widely in many tissues (Kuiper et al. 1997), exposure to estrogenic EEDs may also influence the development of nonreproductive tissues (Meeker 2012). Therefore, detecting environmental estrogens and identifying their sites and mechanism of action during organismal development is of paramount importance.

Standard methods to detect ER activity use yeast and mammalian cell culture assays (Legler et al. 1999; Leskinen et al. 2005; Routledge and Sumpter 1996; Sanseverino et al. 2005) that are limited in their utility because they are not representative of tissue diversity. In addition, although these methods can demonstrate the presence of estrogenic chemicals in environmental samples, they do not address whether chemicals are being absorbed and producing an effect at the organismal level. ER activity assays have been developed for fish and mice; however, most reporter constructs are designed to act in certain tissues exclusively (such as liver or brain) (Brion et al. 2012; Kurauchi et al. 2005) or have used a bioluminescent reporter (such as luciferase) that has limited spatial resolution (Ciana et al. 2003; Legler et al. 2000).

Previously, we developed transgenic zebrafish that specifically report ER transcriptional activity in all tissues of embryos and larvae with single cell resolution (Gorelick and Halpern 2011). The reporter line (5xERE:GFP) contains tandem estrogen response element (ERE) DNA sequences (Gruber et al. 2004) driving green fluorescent protein (GFP) expression. The 5xERE:GFP line serves as a tissue-specific reporter of ER-mediated transcriptional activity following exposure of zebrafish embryos to estrogenic compounds. Exposure to certain purified compounds results in preferential activation of GFP in heart valves, whereas other compounds activate the reporter only in the liver (Gorelick and Halpern 2011). Similar results were reported independently using a 3xERE zebrafish reporter (Lee et al. 2012). We sought to determine whether the ER reporter zebrafish would also be useful in detecting the presence of environmental estrogens and in discovering the basis for the tissue-specific differences in response to estrogens.

## Materials and Methods

*Chemicals.* Estradiol (purity ≥ 98%), bisphenol A (BPA; purity ≥ 99%) and dimethyl sulfoxide (DMSO; purity ≥ 99.9%) were purchased from Sigma-Aldrich (St. Louis, MO). ICI 182,780 (ICI), an ER antagonist; 2,3-*bis*(4-hydroxyphenyl)-propionitrile (DPN) and 4,4´,4´´-(4-propyl-[1*H*]-pyrazole-1,3,5​-triyl)*tris*phenol (PPT), synthetic ER agonists with affinity for human ERα and ERβ, respectively; and 1,3-*bis*(4-hydroxyphenyl)-4-methyl-5​-[4-(2-piperidinylethoxy)phenol]-1*H*-pyrazole dihydrochloride (MPP) and 4-[2-phenyl-5,7-*bis*(trifluoromethyl)pyrazolo[1,5-*a*]pyrimidin-3-yl]phenol (PHTPP), antagonists for ERα and ERβ, respectively, were obtained from Tocris Biosciences (Bristol, UK), with purity of > 99%, except for MPP (purity > 98%). All chemicals were dissolved in DMSO and diluted into dechlorinated fish water for a final DMSO concentration of 0.1%.

*Zebrafish.* Water used to house zebrafish was ultraviolet-light sterilized and circulated through a fluidized bed filtration system (Aquaneering Inc., San Diego, CA). We used three zebrafish strains: the wild-type AB laboratory strain (Walker 1999) and the transgenic strains *Tg(5xERE:GFP)^c262/c262^* and *Tg(5xERE:GFP)^c263^* (Gorelick and Halpern 2011). All work was approved by the Institutional Animal Care and Use Committee of the Carnegie Institution for Science. All animals were treated humanely and with regard for alleviation of suffering.

*RNA* in situ *hybridization*. We used antisense RNA probes corresponding to *esr1* (ERα), *esr2a* (ERβ1), and *esr2b* (ERβ2) as described previously (Gorelick and Halpern 2011). [Note that some previous publications (e.g., Bertrand et al. 2007) referred to the *esr2b* gene as *esr2a* and the *esr2a* gene as *esr2b*.] We also assayed sense RNA probes, but they did not produce signals above background (data not shown). Colorimetric whole-mount *in situ* hybridization was performed on zebrafish embryos and larvae as described previously (Gorelick and Halpern 2011), except 5% dextran (final) was included in the hybridization solution (Lauter et al. 2011). Images were collected using a Zeiss Axioskop microscope equipped with an AxioCam HRc digital camera (Carl Zeiss Microimaging, Thornwood, NJ). Image adjustments and cropping were performed using Photoshop CS5 and InDesign CS5 (both from Adobe Systems Inc., San Jose, CA).

*Water sampling*. To concentrate estrogens over time, passive sampling devices [Polar Organic Chemical Integrative Sampler (POCIS), fabricated at the U.S. Geological Survey (USGS) Columbia Environmental Research Center (Columbia, MO) as described by Alvarez et al. (2004)] were deployed in rivers and streams at 19 locations in the Shenandoah watershed and the Allegheny, Delaware, and Susquehanna Rivers in Virginia and Pennsylvania in April 2010 and remained in place for 31–45 days (see Supplemental Material, Table S1). The Shenandoah and Susquehanna sites are part of an ongoing monitoring and research program to determine the factors involved in fish lesions and mortalities and to assess signs of reproductive endocrine disruption (testicular oocytes and plasma vitellogenin in male bass) observed in these watersheds (Blazer et al. 2010; Reif et al. 2012). The Allegheny and Delaware sites were used as comparisons for the Susquehanna sites in the Pennsylvania emerging contaminants project (Reif et al. 2012). The POCIS devices were deployed during April and May because these months were previously identified as periods of high estrogenicity in the Virginia watershed (Ciparis et al. 2012). After 31–45 days, the sampling devices were retrieved as described by Alvarez (2010), and POCIS membranes were shipped to the USGS Columbia Environmental Research Center for analyte recovery as previously described (Alvarez et al. 2009). Briefly, the POCIS membranes were extracted using 50 mL of 1:1:8 (vol:vol:vol) methanol:toluene:dichloromethane followed by 20 mL ethyl acetate. Extracts were reduced by rotary evaporation, filtered, and composited into two equivalent POCIS samples, thereby increasing the amount of chemical present in each sample to aid in detection.

Samples were resuspended in DMSO and diluted into fish water between 1:100 and 1:4,000 (vol:vol). At 1 day postfertilization (dpf), *Tg(5xERE:GFP)^c262/c262^* embryos were exposed to treated water; they were examined for fluorescent labeling at 3 or 4 dpf. Four embryos were exposed per treatment (see Supplemental Material, Table S2). Embryos were incubated in 24- or 96-well plates at a density of no more than four and two embryos per well, respectively. Exposure occurred under static water conditions, with no water changes during exposure. Embryos were incubated at 28°C under an 18-hr light/6-hr dark cycle.

For discrete water sampling, two sites from the POCIS deployment were selected for follow-up analysis based on results from the initial zebrafish assay. Muddy Creek was selected because samples from that site preferentially activated the reporter in heart valves. Hawksbill Creek was selected because samples from that site exhibited the most intense fluorescence. Water was collected from the Muddy Creek and Hawksbill Creek locations (corresponding to samples 7 and 16 from the POCIS study; see Supplemental Material, Table S1) approximately 1 year after passive sampling to minimize seasonal effects. Samples were extracted with OASIS HLB glass cartridges (Waters Corporation, Milford, MA) as described by Ciparis et al. (2012). The methanol/methanol:dichloromethane eluate was dried under a continuous flow of atmospheric air, resuspended in DMSO, and serially diluted into fish water from 1:500 to 1:10,000 (vol:vol; equivalent to exposing larvae to 5–100 times the concentration found at sampling sites).

For negative controls, a field blank was prepared for each POCIS site and treated identically to POCIS extractions (Alvarez 2010). Briefly, field blanks were stored in airtight containers and transported to the field locations in insulated coolers. During both deployment and retrieval of the passive samplers the lids of the field blank containers were opened and exposed to the surrounding air; this simulated possible exposure to airborne contaminants of the actual deployed sampler. The field blanks were then extracted using the same method as for the deployed sampler. For POCIS samples diluted 1:100 into fish water, the vehicle control was zebrafish incubated in fish water containing 1% DMSO. For other conditions, the vehicle control was zebrafish incubated in fish water containing 0.1% DMSO. For positive controls, zebrafish were incubated in water containing 100 ng/mL estradiol.

Samples were randomly coded so that researchers were blinded to sample identity during zebrafish testing. For the initial screening, GFP fluorescence within live embryos and larvae was visualized using an Olympus MVX10 fluorescent stereomicroscope (Olympus, Center Valley, PA) equipped with a Leica DCF500 digital camera (Leica Microsystems Inc., Buffalo Grove, IL). Images were captured using identical microscope and camera settings. For secondary imaging at higher magnification, embryos and larvae were mounted on bridged coverslips and examined on a Zeiss Axio Imager microscope equipped with an AxioCam HRm digital camera (Carl Zeiss Microimaging, Thornwood, NJ).

*Morpholinos*. To reduce levels of ER protein, 1-cell-stage *Tg(5xERE:GFP)^c262/c262^* embryos were injected with antisense morpholino oligonucleotides targeting the translation start sites of either *esr2a* (5´-ACAT​GGTGAAGG​CGGA​TGAG​TTCA​G) or *esr2b* (5´-AGCT​CATG​CTGG​AGAA​CACA​AGAG​A) (Gene Tools, Philomath, OR). Morpholinos were resuspended in water at 30 μM, and 1–2 nL was injected into each embryo as described by Nasevicius and Ekker (2000). Beginning at 2 dpf, embryos were incubated in 10 μM BPA or vehicle control (0.1% ethanol). At 3 dpf, fluorescence was assayed as described above.

*Yeast ER reporter assay*. To measure estrogen equivalents (relative to 17β-estradiol) of the analytes present in the POCIS extracts, we performed a bioluminescent yeast estrogen screen (BLYES) (Sanseverino et al. 2005) as described previously (Ciparis et al. 2012). All assay plates included a 12-point standard curve consisting of estradiol (2.3 × 10^–11^ to 5.0 × 10^–7^ M) and sample blanks containing minimal media only. Samples, standards, and blanks were run in triplicate. Luminescence was quantified using a SpectraFluor Plus plate reader (Tecan Group Ltd., Durham, NC). A linear calibration curve was created using log_10_ transformations of the five lowest standards (2.3 × 10^–11^ to 2.1 × 10^–10^ M estradiol) and their associated mean luminescence. Concentrations in samples with luminescence above this range were quantified using four points from the linear portion of the dose–response curve (log_10_[estradiol] vs. mean luminescence; 1.2 × 10^–10^ to 1.9 × 10^–9^ M estradiol), extrapolated from these standards, and reported as ng/POCIS estradiol equivalents (E_2_Eq).

## Results

**Figure 1:**
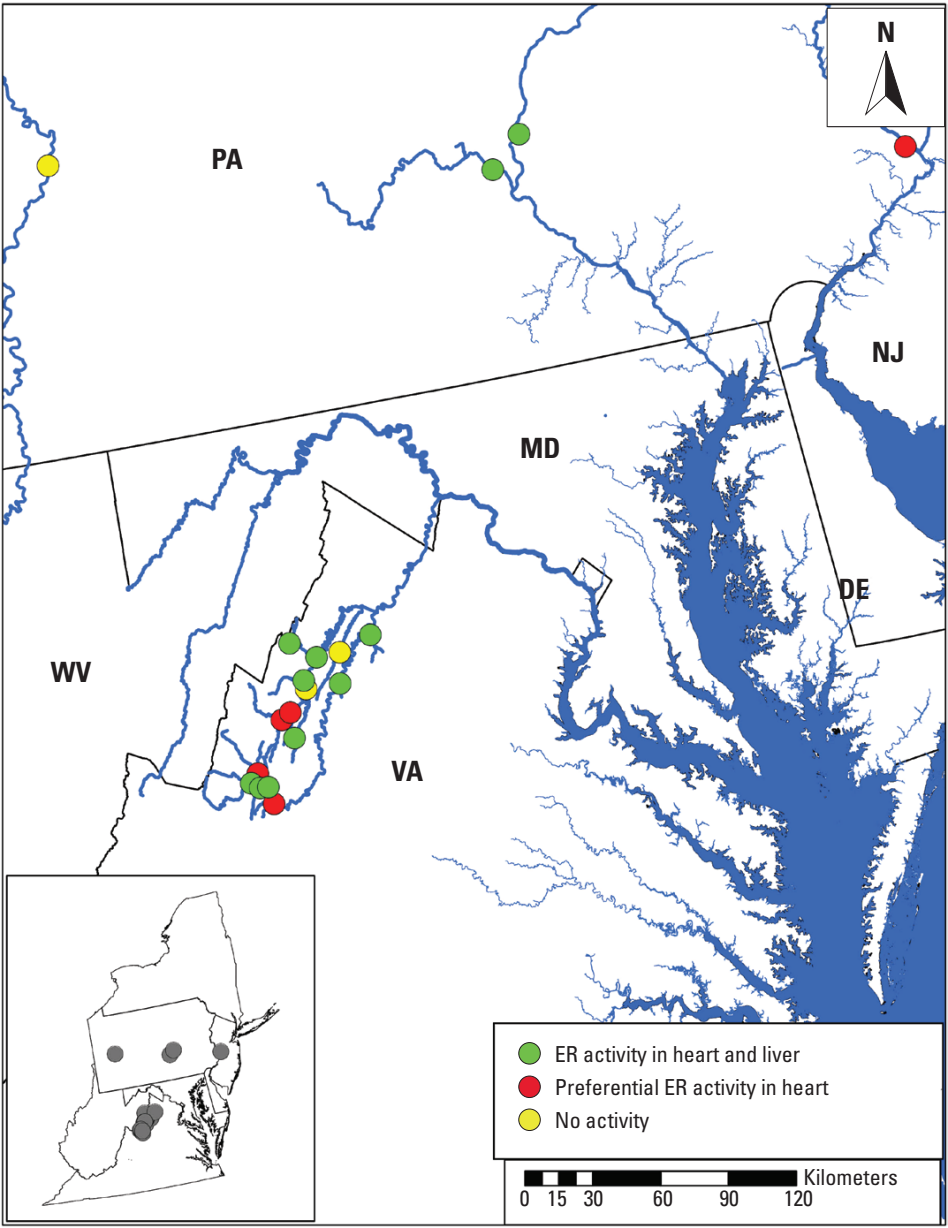
Sites of sample collection (*n* = 19) in April and May of 2010. Each circle represents a sampling site, and the color of the circle indicates the presence or absence of ER activity in the 5xERE:GFP zebrafish reporter after incubation in water containing extracts from sampled water. Abbreviations: DE, Delaware; MD, Maryland; NJ, New Jersey; PA, Pennsylvania; VA, Virginia; WV, West Virginia.

*Environmental estrogens preferentially activate receptors in heart valves.* At 1 dpf, groups of 5xERE:GFP transgenic zebrafish embryos were exposed for 3–4 days to POCIS extracts collected from 19 locations in the Shenandoah River watershed and the Allegheny, Delaware, and Susquehanna Rivers (Figure 1; see also Supplemental Material, Table S1). A surprisingly large number of samples (16) activated the ER reporter in transgenic zebrafish, with 5 samples preferentially inducing GFP labeling of the heart valves (Figure 1; see also Supplemental Material, Table S2 and Movie S1). In embryos exposed to samples 3 (Delaware River; diluted 1:1,000) and 6 (Naked Creek, VA; diluted 1:500), the ER reporter was activated in the heart valves but not the liver (see Supplemental Material, Table S2). Embryos exposed to sample 7 (Muddy Creek, VA) showed activation in both tissues, but with increased sensitivity in the heart valves (1:1,000 dilution) compared with the liver (1:500 dilution).

**Figure 2:**
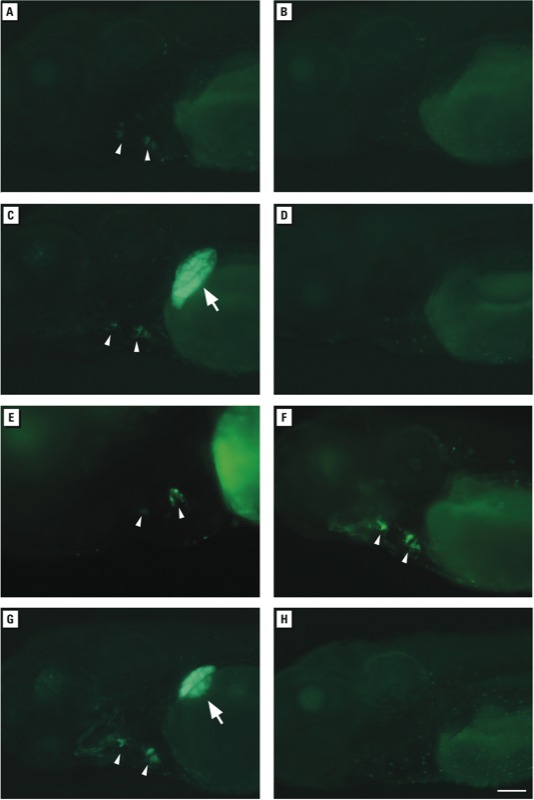
Tissue-specific responses of *Tg(5xERE:GFP)^c262^* zebrafish embryos to environmental estrogens after incubation in water containing extracts from water sampled from the Shenandoah Watershed and nearby rivers. Samples shown in (*A–E*) were collected in 2010 using passive sampling. (*A*) Delaware River, Pennsylvania, sample 3 (diluted 1:1,000). (*B*) Delaware River, sample 3 plus the estrogen receptor antagonist ICI (10 μM). (*C*) Hawksbill Creek, Virginia, sample 16 (diluted 1:1,000). (*D*) Hawksbill Creek, sample 16 plus 10 μM ICI. (*E*) Naked Creek, Virginia, sample 6 (diluted 1:500). (*F*) Hawksbill Creek sample (discrete sampling) collected in 2011 (diluted 1:500). (*G*) Positive control water containing 100 ng/mL estradiol. (*H*) Negative control field blank. Fluorescence was visualized in the liver (arrows) and heart valves (arrow heads) of live larvae at 3 dpf (*F*) or 4 dpf (*A–E,G,H*). All images are lateral views, with anterior to the left and dorsal to the top. Bar = 100 μm.

To confirm that reporter activity was specific for ERs, we exposed embryos to water samples that either activated the reporter in the heart valves alone (samples 3 or 7, diluted 1:1,000) or together with the liver (samples 16 or 18, diluted 1:1,000) in the presence of the ER antagonist ICI (Robertson 2001) (Figure 2). Co-treatment with 10 μM ICI abolished fluorescence in all embryos (Figure 2B,D; see also Supplemental Material, Table S2), indicating that the chemicals in the water were either ER agonists or led to the production of ER agonists in zebrafish. Embryos treated with 100 ng/mL estradiol exhibited robust fluorescence in the heart and liver (Figure 2G), whereas embryos treated with 10 μM ICI alone did not exhibit fluorescence (data not shown), consistent with previous studies (Gorelick and Halpern 2011). Thus, 5xERE:GFP transgenic zebrafish larvae were able to report tissue-specific ER activation of unknown estrogens from passively sampled water.

POCIS sampling provides a time-weighted average of chemical exposure over several weeks, whereas discrete sampling provides a snapshot of chemical exposure at a single point in time. We examined whether the zebrafish reporter was sensitive enough to detect environmental estrogens from single pass collections at the Muddy Creek and Hawksbill Creek locations (samples 7 and 16 from the passive sampling study; see Supplemental Material, Table S1). Approximately 1 year after passive sampling, we collected and concentrated 1 L of water from the same locations. As in the previous findings, water from Hawksbill Creek diluted 1:500 or 1:1,000 activated the reporter in the heart valves (Figure 2F), whereas fluorescence was not observed at greater dilutions (1:5000, 1:10,000) (data not shown; *n* = 20 embryos per dilution per sample). Thus, 5xERE:GFP embryos were able to detect environmental estrogens from water samples collected from the same sites at different times using passive or discrete sampling methods.

To assess the sensitivity of the 5xERE:GFP zebrafish reporter, we compared the responses in zebrafish with those measured using a widely used yeast reporter assay (Balsiger et al. 2010; Leskinen et al. 2005; Sanseverino et al. 2005). Passively sampled water was tested using the BLYES (Sanseverino et al. 2005), which utilizes a yeast strain containing the human *ESR1* (ERα) gene and a tandem ERE that drives an inducible *luxAB* reporter. Every water sample that activated the zebrafish reporter was readily detected using the yeast system (E_2_Eq between 0.76 and 7.98 ng/POCIS; Supplemental Material, Table S2). Moreover, the three water samples that failed to activate the zebrafish reporter exhibited the lowest levels of activity in the yeast assay (< 0.8 E_2_Eq; see Supplemental Material, Table S2).

*Tissue specificity in ER gene expression*. A plausible explanation for the tissue-specific differences in activation of the transgenic reporter by known estrogenic compounds and environmental samples is diversity in ERs. Zebrafish express three ER subtypes, ERα, ERβ1, and ERβ2 (encoded by the genes *esr1*, *esr2a*, and *esr2b*), which, similar to their mammalian orthologues, bind ligands with different affinities *in vitro* (Cosnefroy et al. 2012; Menuet et al. 2002). Previous studies demonstrated that *esr2b* is expressed in the embryonic and larval liver (Bertrand et al. 2007; Gorelick and Halpern 2011). However, there has been no report of ER transcripts or proteins in the developing heart of zebrafish.

**Figure 3:**
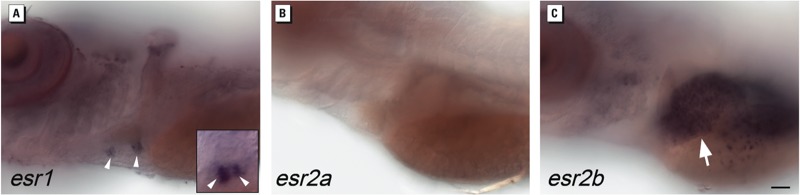
Expression of *esr1*, *esr2a*, and *esr2b* genes in heart and liver detected using whole mount in situ hybridization larvae at 5 dpf. (*A*) esr1 transcripts are present in heart valves (arrow heads) but not in liver. Inset, high magnification ventral view of heart showing labeling of atrioventricular valve leaflets. (*B*) *esr2a* transcripts are not present in heart or liver. (*C*) *esr2b* transcripts are present in liver (arrow) but not in heart valves. All views are lateral views with anterior to the left. Bar = 50 μm.

To determine the presence of ER transcripts in the zebrafish heart, we reexamined expression of ER genes in 3–5 dpf zebrafish using a method for whole-mount *in situ* hybridization with enhanced sensitivity (Lauter et al. 2011). In 5 dpf zebrafish larvae, we observed robust expression of *esr2b* in the liver, consistent with previous studies (Bertrand et al. 2007; Gorelick and Halpern 2011) and discovered that *esr1* is selectively transcribed in the developing valves of the heart (Figure 3). We did not detect *esr2a* transcripts at this stage (Figure 3B). The results demonstrate that different ER subtypes are specifically expressed in the heart and liver. In a previous study we found that the ER ligands BPA and genistein preferentially activated receptors in zebrafish heart valves compared with the liver (Gorelick and Halpern 2011). The differences in ER subtype localization reported here support the idea that BPA and genistein preferentially activate ERα in the heart because they have a higher affinity for this ER subtype.

**Figure 4:**
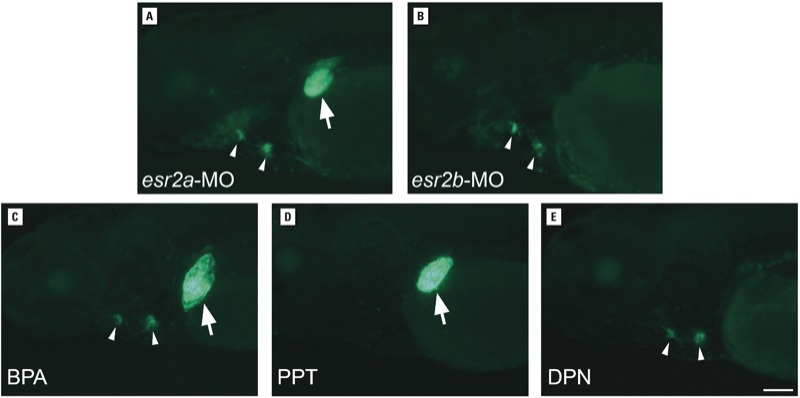
ER subtype-specific activity and tissue-specific response of 5xERE:GFP embryos that were injected with *esr2a* (*A*) or *esr2b* (*B*) antisense morpholino oligonucleotides (MO) to inhibit translation of ERβ1 or ERβ2 proteins. Embryos were exposed to 10 μM BPA at 2 dpf, and fluorescence was visualized a day later. Fluorescence was visualized in the liver (arrows) and heart valves (arrow heads) of live larvae. Embryos injected with *esr2a*‑MO (*A*) exhibited fluorescence in the liver and heart valves, whereas those injected with *esr2b*‑MO (*B*) exhibited fluorescence in heart valves but not liver. (*C–E*) Embryos were exposed to 10 μM BPA or one of the ER subtype-specific agonists (PPT, 100 μM; DPN, 1 μM) at 3 dpf, and fluorescence was visualized a day later. PPT and DPN selectively activated the reporter in liver or heart valves. All images are lateral views, with anterior to the left and dorsal to the top. Bar = 100 μm.

*Selective ER modulation in the heart and liver*. To corroborate the findings of differential gene expression, we used genetic and pharmacological approaches to activate or inhibit ERα, ERβ1, or ERβ2 selectively in 3–4 dpf transgenic zebrafish. On the basis of gene expression, reducing ERβ2 protein levels should reduce ER activity in the liver but not in heart valves. We injected 5xERE:GFP embryos with antisense morpholino oligonucleotides targeting *esr2a* or *esr2b* genes (1–2 nL of 30 μM solution), incubated embryos in 10 μM BPA, and assayed fluorescence. Fluorescence in the liver was reduced in *esr2b*-morphant embryos (Figure 4B) compared with *esr2a*-morphant embryos (Figure 4A), whereas robust labeling of the heart valves was observed in all morphant embryos (Figure 4A,B and Table 1). Embryos exposed to the vehicle alone (0.1% ethanol) exhibited no fluorescence in the liver or heart (data not shown). Attempts to reduce ERα levels using three different morpholinos targeting *esr1* gene translation and RNA splicing were ineffective because *esr1*-morphant embryos exhibited pleiotropic developmental defects such as cardiac edema, a small head, and curved tail (data not shown), suggesting a nonspecific response.

**Table 1:** Results of *esr* morpholino treatment.

Morpholino target	Morpholino dose of 30 μM solution	GFP^+^ heart valves only (%)	GFP^+^ liver only (%)	GFP^+^ heart valves and liver (%)	Embryos (*n*)
*esr2a*	2 nL	0	0	100	18
*esr2b*	1 nL	56	0	44	18
*esr2b*	2 nL	94	0	6	18
One-cell stage 5xERE:GFP embryos were injected with translation blocking morpholinos to reduce ER levels. At 2 dpf, embryos were incubated in 10 μM BPA. Fluorescence was assayed at 3 dpf, and data are presented as the percentage of GFP-positive embryos (GFP^+^) in the indicated tissues.					

To activate ER subtypes selectively, we used the synthetic ER ligands PPT and DPN (Meyers et al. 2001; Stauffer et al. 2000). PPT has higher affinity for human ERα than for ERβ, but DPN has higher affinity for ERβ. We found, however, that 5xERE:GFP zebrafish embryos exposed to 100 μM PPT showed GFP labeling of only the liver, where ERβ2 is produced (*n* = 10; Figure 4D); those exposed to 1 μM DPN showed GFP-labeling of the heart valves, which synthesize ERα (*n* = 20; Figure 4E). Embryos exposed to 10 μM BPA, the positive control, showed GPF labeling of the heart valves and liver (Figure 4C). To inhibit ER subtypes selectively, we exposed zebrafish to selective antagonists designed against human ER subtypes. Treatment of 5xERE:GFP embryos with either the ERα or ERβ antagonists MPP (Sun et al. 2002) or PHTPP (Compton et al. 2004) failed to inhibit reporter activity in any tissue (data not shown). Thus, different ER agonists selectively activated receptors in either the heart valves or the liver of zebrafish larvae, but in a manner opposite to what we expected based on their activation of human ER receptors.

## Discussion

In the present study we observed that 5xERE:GFP reporter zebrafish can detect the tissue-specific effects of environmental estrogens. This represents a significant improvement over traditional detection assays using yeast (Routledge and Sumpter 1996) or cultured cells (Legler et al. 1999), which do not allow comparisons between multiple tissues. Furthermore, testing compounds for ER activity in zebrafish larvae involves the physiologically relevant parameters of absorption, distribution, metabolism, and excretion.

We found a high concordance between responses in zebrafish and in a bioluminescent yeast assay for detection of estrogens from the same environmental samples. This indicates that the whole embryo assay of transgenic zebrafish correlates well with an established and sensitive method (Bergamasco et al. 2011; Ciparis et al. 2012; Sanseverino et al. 2005) for measuring estrogenic compounds in water samples. In addition, the zebrafish reporter revealed a previously unknown tissue and developmental stage for ER signaling, the newly formed heart valves. With the genetic and pharmacological tools available to manipulate zebrafish, transgenic models can be readily applied to detect tissue-specific environmental estrogens and identify their mode of action. Future studies will broaden this approach to report the activity of other environmentally relevant small molecules such as androgens and dioxins.

Although estrogens in discrete and time-integrated passively collected water samples were detectable in 5xERE:GFP zebrafish, our results suggest that estrogen levels vary depending on the sampling method. For example, POCIS extracts prepared from water collected from Muddy Creek in June 2010 activated the reporter preferentially in heart valves, whereas discrete water samples collected the following year did not. Similarly, POCIS extracts from Hawksbill Creek collected in June 2010 activated the reporter in the heart valves and liver, but discrete water samples collected the following year preferentially activated the reporter only in heart valves. These differences are not surprising, however, given the likely daily and seasonal variations in the concentration of environmental estrogens (Ciparis et al. 2012; Martinovic et al. 2008).

An unexpected finding is that that DPN and PPT appear to activate zebrafish ERα and ERβ2, respectively, the opposite of what has been observed for the human ER subtypes (Meyers et al. 2001; Stauffer et al. 2000). One possibility is that zebrafish ERα has greater functional homology to human ERβ. Although zebrafish ERα is most similar to human ERα when comparing the entire protein sequence, similarities between functional domains within each protein are more relevant for predicting functional homology. For example, in the N-terminal AF-1 domain that regulates transcriptional activation (also referred to as the A/B domain) (Metzger et al. 1995), zebrafish ERα is more similar to human ERβ (13.2%) than to human ERα (8.4%) (Menuet et al. 2002). Low sequence homology (< 15% identity) between the AF-1 domains from human and zebrafish ERs makes it difficult to predict functional homology between subtypes with accuracy. Furthermore, studies using chimeric ER proteins from rainbow trout and humans suggest that, despite low sequence homology, ER domains from different species may function similarly and interact with the same transcription factors (Petit et al. 2000). It is therefore not surprising that agonists might show altered affinities for ERs in species as diverse as fish and humans.

Although ER-subtype–selective agonists (DPN and PPT) designed against human ERs were effective in zebrafish, selective antagonists designed against human ER subtypes were not. These *in vivo* results are consistent with those obtained in cultured cells expressing zebrafish ERs, where MPP and PHTPP also failed to inhibit ERE-dependent reporter activity induced by 17α-ethynylestradiol (Notch and Mayer 2011). Together, these data suggest that MPP and PHTPP do inhibit zebrafish ERs.

The environmental estrogenic compound(s) capable of activating the zebrafish reporter with tissue specificity remains to be identified. The low levels of known estrogens in water samples make this a challenging endeavor, requiring sequential rounds of HPLC fractionation for purification and mass spectrometry for identification. However, the small size and transparency of zebrafish embryos are advantageous for rapid, high-throughput screening of fractions for tissue-specific ER activity. Ultimately, it will be possible to identify unknown EEDs that affect estrogen signaling, their sites of action, and effects on embryonic development.

The activation of ERs in heart valves during development leads to the intriguing hypothesis that estrogen signaling influences valve formation. In humans, the occurrence of heart valve abnormalities differs between the sexes, which could be due to sex differences in estrogen levels. Bicuspid aortic valve defects, where the aortic valve develops two leaflets instead of three, are four times more prevalent in men than in women (Warnes 2008). Because ERs are ligand-dependent transcription factors, it will be important to identify which genes are directly regulated by estrogens and to test whether they are important for cell migration or proliferation of valve precursors. Exposure to environmental endocrine-disrupting compounds that mimic or inhibit endogenous estrogens *in utero* is associated with adverse health effects (Soto and Sonnenschein 2010), with the potentially unanticipated consequence of causing heart valve malformations.

## Supplemental Material

(94 KB) PDF

(7.7 MB) MOV
