# Supplementary material for: Transgenic Zebrafish Reveal Tissue-Specific Differences in Estrogen Signaling in Response to Environmental Water Samples
Source: Environ Health Perspect. 2014 Jan 14;122(4):356–62. doi: 10.1289/ehp.1307329 (PMC3984228; doi:10.1289/ehp.1307329)
Supplement: (94 KB) PDF [file ehp.1307329.s001.pdf]

Environmental Health Perspectives 10.1289/ehp.1307329

Supplemental Material
Transgenic Zebrafish Reveal Tissue-Specific Differences in Estrogen Signaling in Response to Environmental Water Samples

Daniel A. Gorelick, Luke R. Iwanowicz, Alice L. Hung, Vicki S. Blazer, and Marnie E. Halpern

Table of Contents

Supplemental Material, Table S1 p. 2

Supplemental Material, Table S2 p. 3

Supplemental Material, Movie S1 p. 5

Supplemental Material, Table S1. 2010 Susquehanna, Delaware, Allegheny and Shenandoah River Watershed Passive Sampling Study.

Sample ID	Deployment date	Retrieval date	Total days	Location #	Location	N	W
1	4/21/10	6/1/10	41	SUS 5	Juniata River, Newport, Pennsylvania	40.4910	-77.0995
2	4/21/10	6/1/10	41	SUS 6	Susquehanna River at Mahantango Creek, Browns Island, Pennsylvania	40.6515	-76.9228
3	4/19/10	6/3/10	45	DE-1	Delaware River, Yardley, Pennsylvania	40.2514	-74.8414
4	4/28/10	6/7/10	40	AL-1	Allegheny River, Kittanning, Pennsylvania	40.8138	-79.5252
5	5/12/10	6/14/10	33	PSS 16	Cooks Creek, Virginia	38.3734	-78.9374
6	5/12/10	6/15/10	34	PSS 09	Naked Creek, Virginia	38.3088	-78.9226
7	5/13/10	6/15/10	33	PSS 15	Muddy Creek, Virginia	38.4308	-78.9806
8	5/13/10	6/15/10	33	PSS 10	Briery Branch, Virginia	38.3995	-79.0232
9	5/13/10	6/14/10	32	PSS 12	Long Glade Creek, Virginia	38.3773	-78.9806
10	5/13/10	6/14/10	32	PSS 11	Smith Creek (upstream), Virginia	38.5382	-78.7505
11	5/13/10	6/14/10	32	PSS 13	Linville Creek, Virginia	38.6141	-78.8004
12	5/13/10	6/14/10	32	PSS 14	Long Meadow Run, Virginia	38.6339	-78.7484
13	5/13/10	6/14/10	32	PSS 08	Smith Creek (downstream), Virginia	38.7116	-78.6416
14	5/13/10	6/14/10	32	PSS 06	Mill Creek, Virginia	38.7433	-78.6450
15	5/14/10	6/14/10	31	PSS 02	Gooney Run, Virginia	38.8669	-78.2477
16	5/14/10	6/14/10	31	PSS 07	Hawksbill Creek, Virginia	38.7083	-78.4564
17	5/14/10	6/15/10	32	PSS 03	Passage Creek, Virginia	38.8238	-78.4281
18	5/14/10	6/15/10	32	PSS 05	Stony Creek (downstream), Virginia	38.8210	-78.5550
19	5/14/10	6/15/10	32	PSS 04	Stony Creek (upstream), Virginia	38.8912	-78.6843

Supplemental Material, Table S2. Reporter activity in 3-4 day old 5xERE:GFP zebrafish in response to 2010 Susquehanna, Delaware, Allegheny and Shenandoah passive samples.

Sample ID	Location	E2Eq[a]	Dilution	Reporter activity	Total embryos (N)[b]
1	SUS 5	2.94	1:100	lethal	4
1	SUS 5	2.94	1:500	GFP heart valves, liver	5
1	SUS 5	2.94	1:1000	GFP-negative	15
2	SUS 6	3.13	1:100	lethal	4
2	SUS 6	3.13	1:500	GFP heart valves, liver	5
2	SUS 6	3.13	1:1000	GFP-negative	15
3[c]	DE-1	1.58	1:100	lethal	4
3[c]	DE-1	1.58	1:1000	GFP heart valves	4
3[c]	DE-1	1.58	1:1000	GFP heart valves, liver	10
3[c]	DE-1	1.58	1:1000 + 10 μM ICI 182,780	GFP-negative	5
4[d]	AL-1	0.78	1:100	lethal	4
4[d]	AL-1	0.78	1:500	GFP-negative	5
4[d]	AL-1	0.78	1:1000	GFP-negative	15
5	PSS 16	3.79	1:100	lethal	4
5	PSS 16	3.79	1:1000	GFP heart valves, liver	15
5	PSS 16	3.79	1:2000	GFP-negative	5
6[c]	PSS 09	2.55	1:100	lethal	4
6[c]	PSS 09	2.55	1:500	GFP heart valves	30
6[c]	PSS 09	2.55	1:1000	GFP-negative	15
7[c]	PSS 15	2.86	1:100	lethal	4
7[c]	PSS 15	2.86	1:500	GFP heart valves, liver	5
7[c]	PSS 15	2.86	1:1000	GFP heart valves	15
7[c]	PSS 15	2.86	1:1000	GFP-negative	25
7[c]	PSS 15	2.86	1:1000 + 10 μM ICI 182,780	GFP-negative	5
8	PSS 10	1.18	1:100	GFP heart valves, liver	9
8	PSS 10	1.18	1:500	GFP-negative	5
8	PSS 10	1.18	1:1000	GFP-negative	15
9	PSS 12	5.26	1:100	lethal	4
9	PSS 12	5.26	1:500	GFP heart valves, liver	5
9	PSS 12	5.26	1:1000	GFP heart valves, liver	10
9	PSS 12	5.26	1:1000	GFP-negative	10
10	PSS 11	2.36	1:500	GFP heart valves	1
10	PSS 11	2.36	1:500	GFP heart valves, liver	3
10	PSS 11	2.36	1:1000	GFP-negative	15
11[c]	PSS 13	2.85	1:1000	GFP heart valves, liver	10
11[c]	PSS 13	2.85	1:2000	GFP heart valves	6
11[c]	PSS 13	2.85	1:2000	GFP-negative	23
11[c]	PSS 13	2.85	1:4000	GFP-negative	5
12[c]	PSS 14	5.41	1:1000	GFP heart valves, liver	10
12[c]	PSS 14	5.41	1:2000	GFP heart valves	13
12[c]	PSS 14	5.41	1:2000	GFP heart valves, liver	1
12[c]	PSS 14	5.41	1:2000	GFP-negative	15
12[c]	PSS 14	5.41	1:4000	GFP-negative	5
13[d]	PSS 08	0.76	1:500	GFP-negative	5
13[d]	PSS 08	0.76	1:1000	GFP-negative	15

Sample ID	Location	E2Eq[a]	Dilution	Reporter activity	Total embryos (N)[b]
14	PSS 06	4.19	1:500	GFP liver	1
14	PSS 06	4.19	1:500	GFP heart valves, liver	3
14	PSS 06	4.19	1:1000	GFP-negative	15
15	PSS 02	1.28	1:500	GFP heart valves, liver	5
15	PSS 02	1.28	1:1000	GFP-negative	15
16	PSS 07	7.98	1:1000	GFP heart valves, liver	15
16	PSS 07	7.98	1:2000	GFP heart valves, liver	5
16	PSS 07	7.98	1:4000	GFP-negative	5
16	PSS 07	7.98	1:1000 + 10 μM ICI 182,780	GFP-negative	5
17[d]	PSS 03	0.54	1:500	GFP-negative	5
17[d]	PSS 03	0.54	1:1000	GFP-negative	10
18	PSS 05	3.06	1:1000	GFP heart valves, liver	15
18	PSS 05	3.06	1:2000	GFP-negative	5
18	PSS 05	3.06	1:1000 + 10 μM ICI 182,780	GFP-negative	5
19	PSS 04	0.82	1:500	GFP liver	1
19	PSS 04	0.82	1:500	GFP heart valves, liver	3
19	PSS 04	0.82	1:500	GFP-negative	25
19	PSS 04	0.82	1:1000	GFP-negative	15

[a]Estradiol equivalents (ng/POCIS) from bioluminescent yeast estrogen receptor reporter assay (see “Methods” for details). [b]Indicates total number of embryos with indicated phenotype. [c]Indicates samples that preferentially activated the zebrafish reporter in heart valves. [d]Indicates samples that failed to activate the zebrafish reporter.

Supplemental Material, Movie S1. (NOTE: This movie is available as a separate Supplemental Material file that may be downloaded from the *EHP* website.) Estrogen receptors in heart valves respond to environmental estrogens. *Tg(5xERE:GFP)*$^{c262/c262}$ larvae were exposed to POCIS sample 16 (Hawksbill Creek) diluted 1:500 at 2 days post fertilization. The following day, time-lapse images of GFP fluorescence in live larvae were captured for 30 seconds totaling 56 frames, 10 millisecond exposure per frame using a 20X / 0.75 NA objective. The movie plays at 5 frames per second. Note the fluorescence in the aortic and atrioventricular valves. Lateral view; anterior to the left, dorsal to the top.
